# Supplementary material for: Exaptation of ancestral cell-identity networks enables C4 photosynthesis
Source: Nature. 2024 Nov 20;636(8041):143–50. doi: 10.1038/s41586-024-08204-3 (PMC11618092; doi:10.1038/s41586-024-08204-3)
Supplement: Supplementary file 1 — Reporting Summary [file 41586_2024_8204_MOESM1_ESM.pdf]

nature portfolio

Corresponding author(s): Julian Hibberd, Joseph Ecker and Leonie Luginbuehl

Last updated by author(s): Sep 10, 2024

Reporting Summary

Nature Portfolio wishes to improve the reproducibility of the work that we publish. This form provides structure for consistency and transparency in reporting. For further information on Nature Portfolio policies, see our Editorial Policies and the Editorial Policy Checklist.

Statistics

For all statistical analyses, confirm that the following items are present in the figure legend, table legend, main text, or Methods section.

n/a	Confirmed	
☐	☒	The exact sample size (*n*) for each experimental group/condition, given as a discrete number and unit of measurement
☐	☒	A statement on whether measurements were taken from distinct samples or whether the same sample was measured repeatedly
☐	☒	The statistical test(s) used AND whether they are one- or two-sided<br>*Only common tests should be described solely by name; describe more complex techniques in the Methods section.*
☐	☒	A description of all covariates tested
☒	☐	A description of any assumptions or corrections, such as tests of normality and adjustment for multiple comparisons
☒	☐	A full description of the statistical parameters including central tendency (e.g. means) or other basic estimates (e.g. regression coefficient) AND variation (e.g. standard deviation) or associated estimates of uncertainty (e.g. confidence intervals)
☒	☐	For null hypothesis testing, the test statistic (e.g. *F*, *t*, *r*) with confidence intervals, effect sizes, degrees of freedom and *P* value noted<br>*Give P values as exact values whenever suitable.*
☒	☐	For Bayesian analysis, information on the choice of priors and Markov chain Monte Carlo settings
☒	☐	For hierarchical and complex designs, identification of the appropriate level for tests and full reporting of outcomes
☐	☒	Estimates of effect sizes (e.g. Cohen's *d*, Pearson's *r*), indicating how they were calculated

Our web collection on statistics for biologists contains articles on many of the points above.

Software and code

Policy information about availability of computer code

Data collection	BD Influx Sortware v1.2.0.142 (flow cytometry). Illumina MiSeq control software v3.1.0.13 and NovaSeq 6000 control software v1.6.0/RTA v3.4.4 (sequencing).
Data analysis	System: R version 4.2.1, Python 3.10.5<br>Mapping and Assembly: BSgenome 1.64.0, cellranger-3.0.2, cellranger-arc-2.0.1,<br>Anaylsis: ggplot2 3.4.0, Seurat 4.3.0, Signac 1.9.0, EDASeq 2.30.0, DESeq2 1.36.0<br>Other code and required packages can be found at https://github.com/joey1463/C3-C4.git

For manuscripts utilizing custom algorithms or software that are central to the research but not yet described in published literature, software must be made available to editors and reviewers. We strongly encourage code deposition in a community repository (e.g. GitHub). See the Nature Portfolio guidelines for submitting code & software for further information.

Data

Policy information about availability of data

All manuscripts must include a data availability statement. This statement should provide the following information, where applicable:

- Accession codes, unique identifiers, or web links for publicly available datasets
- A description of any restrictions on data availability
- For clinical datasets or third party data, please ensure that the statement adheres to our policy

Raw and processed data, including assembled atlases, have been deposited at GEO and are publicly available (GSE248919). Chlorophyll measurement raw data have been deposited at Mendeley. (doi: 10.17632/6xmsdg9xcr.1). R scripts used for analyses presented in this manuscript are published on github at https://github.com/joey1463/C3-C4.git.

Research involving human participants, their data, or biological material

Policy information about studies with human participants or human data. See also policy information about sex, gender (identity/presentation), and sexual orientation and race, ethnicity and racism.

Reporting on sex and gender	not applicable
Reporting on race, ethnicity, or other socially relevant groupings	not applicable
Population characteristics	not applicable
Recruitment	not applicable
Ethics oversight	not applicable

Note that full information on the approval of the study protocol must also be provided in the manuscript.

Field-specific reporting

Please select the one below that is the best fit for your research. If you are not sure, read the appropriate sections before making your selection.

☒ Life sciences ☐ Behavioural & social sciences ☐ Ecological, evolutionary & environmental sciences

For a reference copy of the document with all sections, see nature.com/documents/nr-reporting-summary-flat.pdf

Life sciences study design

All studies must disclose on these points even when the disclosure is negative.

Sample size	No sample size calculation was performed. The number of nuclei we sequenced per time point allowed us enough coverage to annotate cell types and perform required statistical analyses to detect differentially expressed genes and accessible chromatin.
Data exclusions	No significant data was excluded from our study. Only genes with low read counts, or nuclei with low UMIs, were removed during normal processing of single-nuclei sequencing data.
Replication	Single nuclei sequencing data presented in this study was replicated in the following ways:<br>- For our assay of transcriptional responses to photomorphogenesis, multiple time points were assayed (9 time points).<br>- We sequenced tissue resulting from this time course using both 10X and sci-RNA-seq3 using different plant populations (technical replication, biological replication).<br>- For our multiomic assay, we replicated these with 2 distinct biological replicates and 2-4 technical replicates (technical replication, biological replication). All replication events were successful.<br>Validation experiments in this study were replicated in the following ways:<br>- For our transactivation assay testing DOF transcription factor activity, we performed the experiment 3 times independently with similar results, each with 4 biological replicates. We report only one experiment.<br>- For our GUS reporter of the minimal SIR reporter, we performed 52 independent transformation events. Resulting data for all independent lines are presented.<br>- For chlorophyll measurements, we performed 3 biological replicates, and performed the experiment 3 times independently with similar results.
Randomization	Plant seedlings were randomized in growth chamber. Additionally, random seedling samples (n > 10) were taken at each time point observed. Random subpopulations of nuclei were selected either through FACS sorting or random passage through the sci-RNA-seq3 workflow.
Blinding	Blinding was not relevant in our study. Specifically, photomorphogenesis will turn white tissue visibly green. Thus, throughout our experimental protocol it is possible to identify which sample was light treated.

Reporting for specific materials, systems and methods

We require information from authors about some types of materials, experimental systems and methods used in many studies. Here, indicate whether each material, system or method listed is relevant to your study. If you are not sure if a list item applies to your research, read the appropriate section before selecting a response.

Materials & experimental systems

n/a	Involved in the study	
☒	☐	Antibodies
☒	☐	Eukaryotic cell lines
☒	☐	Palaeontology and archaeology
☒	☐	Animals and other organisms
☒	☐	Clinical data
☒	☐	Dual use research of concern
☐	☒	Plants

Methods

n/a	Involved in the study	
☒	☐	ChIP-seq
☒	☐	Flow cytometry
☒	☐	MRI-based neuroimaging

Dual use research of concern

Policy information about dual use research of concern

Hazards

Could the accidental, deliberate or reckless misuse of agents or technologies generated in the work, or the application of information presented in the manuscript, pose a threat to:

No	Yes	
☒	☐	Public health
☒	☐	National security
☒	☐	Crops and/or livestock
☒	☐	Ecosystems
☒	☐	Any other significant area

Experiments of concern

Does the work involve any of these experiments of concern:

No	Yes	
☒	☐	Demonstrate how to render a vaccine ineffective
☒	☐	Confer resistance to therapeutically useful antibiotics or antiviral agents
☒	☐	Enhance the virulence of a pathogen or render a nonpathogen virulent
☒	☐	Increase transmissibility of a pathogen
☒	☐	Alter the host range of a pathogen
☒	☐	Enable evasion of diagnostic/detection modalities
☒	☐	Enable the weaponization of a biological agent or toxin
☒	☐	Any other potentially harmful combination of experiments and agents

Plants

Seed stocks

We used wild type Oryza sativa spp Japonica cultivar Kitaake, and Sorghum Bicolor BTx623

Novel plant genotypes

Oryza sativa spp Japonica cultivar Kitaake was transformed with a construct tagging bundle sheath nuclei with mTurquoise2. Transformation method was agrobacterium mediated rice transformation. We analyzed 5 independent transgenic single copy lines. Experiments were performed homozygous T2 and T3 seeds. Additionally,
Oryza sativa spp Japonica cultivar Kitaake was transformed with a construct tagging bundle sheath nuclei with the minimal sulfite reductase (SIR) promoter driving GUS expression. Here, 23 and 29 independent T0 plants for two different construct types were analyzed.

Authentication

The presence of the transgene was confirmed using southern blots, PCR and confocal microscopy.
