# Supplementary material for: Exaptation of ancestral cell-identity networks enables C4 photosynthesis
Source: Nature. 2024 Nov 20;636(8041):143–50. doi: 10.1038/s41586-024-08204-3 (PMC11618092; doi:10.1038/s41586-024-08204-3)
Supplement: Supplementary file 3 — Supplementary Tables 1–13. [file 41586_2024_8204_MOESM3_ESM.zip › 2024-01-00217C-s3/2024-01-00217C-SupplementaryTablesGuide.docx]

**Supplementary Table 1:** List of marker genes used for cell type annotation and specific markers for each cluster identified in the de-etiolation single nuclei RNA-seq dataset in rice and sorghum.

**Supplementary Table 2:** List of significant marker genes for each cluster within bundle sheath marker line single nuclei experiment.

**Supplementary Table 3:** Gene names that hold cell type specific accessible chromatin within +/- 2000 bp of TSS.

**Supplementary Table 4:** Normalized expression levels of sorghum bundle sheath marker genes with different patterns of gene expression related to the rice bundle sheath marker genes.

**Supplementary Table 5:** Marker genes for each cell type in rice and sorghum and names and GO enrichment of orthologous marker genes found either in mesophyll/guard cells or the bundle sheath of rice and sorghum respectively.

**Supplementary Table 6:** List of orthologous gene pairs that are partitioned between the mesophyll and bundle sheath in rice and sorghum.

**Supplementary Table 7:** Number of conserved partitioned genes between cell type pairs and patterns of partitioning among higher order orthogroups.

**Supplementary Table 8:** List of genes significantly differentially expressed in response to light.

**Supplementary Table 9**: Normalized expression counts for genes involved in photosynthetic processes.

**Supplementary Table 10:** *Cis*-regulatory motifs enriched in a cell type restricted manner.

**Supplementary Table 11:** Enriched *cis*-regulatory motifs in homologs of bundle sheath specific genes in different *Poaceae* species.

**Supplementary Table 12:** *Cis*-regulatory motifs enriched in response to light within a cell type.

**Supplementary Table 13:** Differentially partitioned genes in rice mesophyll and sorghum bundle sheath and corresponding enriched motifs and DOF motif frequencies.
