# Supplementary material for: Exaptation of ancestral cell-identity networks enables C4 photosynthesis
Source: Nature. 2024 Nov 20;636(8041):143–50. doi: 10.1038/s41586-024-08204-3 (PMC11618092; doi:10.1038/s41586-024-08204-3)
Supplement: Supplementary file 3 — Supplementary Tables 1–13. [file 41586_2024_8204_MOESM3_ESM.zip › 2024-01-00217C-s3/Supplementary Information References Updated_ESM.docx]

**Supplementary Information References**

66. Tsutsumi, K., Taniguchi, Y., Kawasaki, M., Taniguchi, M. & Miyake, H. Expression of photosynthesis-related genes during the leaf development of a C_3_ plant rice as visualized by In Situ hybridization. *Plant Prod Sci* **9**, 232–241 (2006).

67. Kasai, K. *et al.* Differential expression of three plastidial sigma factors, OsSIG1, OsSIG2A, and OsSIG2B, during leaf development in rice. *Biosci Biotechnol Biochem* **68**, 973–977 (2004).

68. Song, S. *et al.* OsFTIP1-mediated regulation of florigen transport in rice is negatively regulated by the ubiquitin-like domain kinase OsUbDKγ4. *Plant Cell* **29**, 491–507 (2017).

69. Bourdenx, B. *et al.* Overexpression of Arabidopsis ECERIFERUM1 promotes wax very-long-chain alkane biosynthesis and influences plant response to biotic and abiotic stresses. *Plant Physiol* **156**, 29–45 (2011).

70. Mahroug, S., Courdavault, V., Thiersault, M., St-Pierre, B. & Burlat, V. Epidermis is a pivotal site of at least four secondary metabolic pathways in Catharanthus roseus aerial organs. *Planta* **223**, 1191–1200 (2006).

71. Funk, V., Kositsup, B., Zhao, C. & Beers, E. P. The Arabidopsis xylem peptidase XCP1 is a tracheary element vacuolar protein that may be a papain ortholog. *Plant Physiol* **128**, 84–94 (2002).

72. Min, M. K. *et al.* Two clade A phosphatase 2Cs expressed in guard cells physically interact with abscisic acid signaling components to induce stomatal closure in rice. *Rice* **12**, 1–13 (2019).

73. Nguyen, T. H. *et al.* A dual role for the OsK5.2 ion channel in stomatal movements and K+ loading into Xylem Sap. *Plant Physiol* **174**, 2409–2418 (2017).

74. Matsukura, C. A. *et al.* Sugar uptake and transport in rice embryo. Expression of companion cell-specific sucrose transporter (OsSUT1) induced by sugar and light. *Plant Physiol* **124**, 85–93 (2000).

75. Scofield, G. N. *et al.* The role of the sucrose transporter, OsSUT1, in germination and early seedling growth and development of rice plants. *J Exp Bot* **58**, 483–495 (2007).

76. Zhao, J. *et al.* ABC transporter OsABCG18 controls the shootward transport of cytokinins and grain yield in rice. *J Exp Bot* **70**, 6277–6291 (2019).

77. Döring, F., Streubel, M., Bräutigam, A. & Gowik, U. Most photorespiratory genes are preferentially expressed in the bundle sheath cells of the C_4_ grass *Sorghum bicolor*. *J Exp Bot* **67**, 3053–3064 (2016).
